# Arthroscopically and manually minced cartilage demonstrates lower cell viability and lower proteoglycan deposition compared to isolated chondrons and chondrocytes

**DOI:** 10.1016/j.ocarto.2026.100843

**Published:** 2026-06-17

**Authors:** Dunja Scheepmaker, Christopher V. Nagelli, Katherine L. Lydon, Lucienne A. Vonk, Aaron J. Krych, Roel JH. Custers, Daniel BF. Saris, Jasmijn V. Korpershoek

**Affiliations:** a Department of Orthopedic Surgery, Mayo Clinic, Rochester, MN, United States of America; b Department of Orthopedic Surgery, University Medical Center Utrecht, Utrecht, the Netherlands

**Keywords:** Minced cartilage, chondrons, chondrocytes, cell viability, Proteoglycan deposition

## Abstract

**Objective:**

Autologous minced cartilage offers a single-stage, low-cost alternative to cell therapies for treatment of chondral defects. It is often derived from debrided defects or loose bodies, then minced for reimplantation. However, there is a paucity of knowledge on how mincing cartilage impacts viability and extracellular matrix deposition compared to quality-controlled cell therapies. The purpose is therefore to better characterize minced cartilage by comparing different mincing techniques to isolated cells.

**Methods:**

Cartilage samples were obtained from fresh human osteochondral allografts (JRF Ortho) and divided into: chondral allograft biopsy (control), manually minced cartilage, arthroscopically minced cartilage, isolated chondrons, and isolated chondrocytes. Samples were embedded in fibrin gels and cultured for 28 days. Staining for viability and histology for collagen and proteoglycan components were performed. Cartilage samples derived from debrided defects or loose bodies were evaluated for sulphated glycosaminoglycans (sGAGs), DNA, and wet weight.

**Results:**

At 28 days, viability of chondrons (93.7 ± 2.9%), chondrocytes (94 ± 22%) and manually minced cartilage (79.8 ± 16.2%) were significantly higher than arthroscopically minced cartilage (56.6 ± 15.4%). Over time, isolated cells showed proteoglycan and type II collagen deposition, while minced cartilage groups showed reduced proteoglycan content on histology. Clinical cartilage samples varied >10-fold in wet weight, total DNA, sGAGs/weight, and DNA/weight.

**Conclusion:**

This study showed isolated cells have consistently high viability and deposit more proteoglycans over time compared to both minced cartilage groups. Samples typically used for clinical application of minced cartilage can vary over 10-fold in cellularity and proteoglycan content. As consequences for treatment effect are unknown, implementation in clinical practice without quality regulation is not recommended.

## 1 Introduction

Articular cartilage defects are common and are reported in up to 66% of arthroscopies yearly [1,2]. Since articular cartilage has a limited capacity for self-repair due to its avascular nature and low chondrocyte activity, surgical intervention is the standard of care for symptomatic lesions. Currently, multiple cartilage repair techniques to treat defects exist. Microfracture is indicated for small cartilage defects and autologous chondrocyte implantation (ACI) for large cartilage defects [3]. Cell-based therapies such as ACI and matrix-assisted ACI (MACI) have been used in clinics for the last 30 years, are regulated as a drug, and held to high quality control standards. Over 500 patients have been treated with ACI or MACI in quality controlled randomized clinical trials and have shown favourable long-term results [4,5]. However, these treatments involve two surgical procedures, high costs, and a delay in returning to activity and rehabilitation [4,5]. New clinical trials for single-stage cell-based therapies using chondrons, such as IMPACT and RECLAIM, show promising results [6,7].

Minced cartilage is increasingly used to treat cartilage defects in the knee as it provides a single-stage alternative with easy logistics and can be used on different defect sizes. The technique involves taking cartilage from debrided defects or loose bodies, followed by mincing and reimplantation, sometimes combined with a type of scaffold or sealants such as fibrin gel or PRP gel [8]. However, minced cartilage is not regulated like drugs or medical implants, therefore reimplantation of minced cartilage occurs without standardized quality control. Furthermore, no clinical development is required such as (comparative) efficacy studies and tracking of adverse events. Current clinical evidence is limited to two RCTs together including little over 100 patients and without reporting quality and quantity of minced cartilage applied [9,10].

In vitro, cartilage minced arthroscopically or with a scalpel demonstrated extracellular matrix (ECM) production up to 6 weeks [11,12]. Prior studies on chondrocyte viability in minced cartilage demonstrated variable results. Arthroscopic mincing of cartilage seems to decrease viability compared to mincing using a scalpel or a control [[12], [13], [14], [15], [16]]. Furthermore, it is unclear to what extent new ECM is formed in minced cartilage as most studies do not correct for the ECM present in cartilage fragments at baseline [11,15,17,18]. Lastly, the level of variability in cell viability, quantity, and tissue composition in clinical samples in unclear.

The aim of this study is therefore to systematically compare viability, tissue formation, and proliferation of isolated chondrons and chondrocytes to arthroscopically and manually minced cartilage. This set-up includes a representation of MACI/ACI [4,5], which are based on isolated and expanded chondrocytes and is currently the standard of care for large cartilage defects, and IMPACT/RECLAIM [6,7], which are cartilage cell transplantations based on autologous chondrons and allogeneic MSCs and currently under clinical development. Furthermore, we aim to assess the quantity and quality of cartilage that is used as starting material for direct clinical application of minced cartilage.

## 2 Methods

A. Healthy cartilage samples

### 2.1 Tissue collection

To allow comparison between groups, all groups were compared within each donor. Healthy human osteochondral allograft tissue was used because the donor age is within the same range as patients that receive treatment of cartilage defects, and it provides sufficient tissue to compare all groups within each donor. Cartilage samples were obtained from leftover fresh human osteochondral allografts (JRF Ortho, Centennial, CO, USA). The storage times were: donor 5 and 6, 17 days; donor 7, 9, 11 and 13, 16 days; donor 8, 21 days; donor 10, 9 days; and donor 12, 11 days (5 male, 4 female, age 13–32). Minimally four donors were included per analysis and extra donors when available. We used four technical replicates per group to ensure sufficient and representative data was collected per group per donor. Five groups were compared: (1) chondral allograft control (procured with a 4 mm biopsy), (2) manually minced cartilage (minced using a scalpel), (3) arthroscopically minced cartilage (using a shaver), (4) isolated chondrons (digested overnight with dispase (0.15%), Gibco (Waltham, MA, USA) and collagenase type 2 (0.1%, Worthington (Lakewood, NJ, USA))), and (5) isolated chondrocytes (digested overnight with pronase (0.2%) (Sigma-Aldrich, St. Louis, MO, USA), and collagenase type 2 (0.075% (Worthington, Lakewood, N, USA)) [19]. Chondrons and chondrocytes were cultured as 250.000 cells in 100 μL to represent the concentration of cells used in ACI. All samples were encapsulated in fibrin gels (1:15 fibrinogen, 1:50 thrombin) (TISSEEL™, Baxter Healthcare Corp, Deerfield, IL, USA) and cultured for 28 days total in growth-factor free chondropermissive medium (DMEM, ThermoFisher Scientific, Waltham, MA, USA) with 2% ITS-X (Life Technologies, Waltham, MA, USA), 1% penicillin/streptomycin (Gibco, Waltham, MA, USA), 4 g/L human serum albumin (Fujifilm Irvine Scientific, Santa Ana, CA, USA), 1% ascorbic acid 2-phosphate (Sigma-Aldrich, St. Louis, MO, USA).

### 2.2 Cell viability

To assess viability, samples were stained with Calcein AM and ethidium homodimer-1 (Invitrogen, Carlsbad, CA, USA) and imaged using fluorescence microscopy. Viability was defined as percentage of living cells out of total cells. Images were captured by Zeiss Zen Microscopy Software (RRID: SCR_013672) and analyzed using ImageJ software [20]. Viability was assessed at 4 timepoints: days 0, three, seven, and 28. The number of donors included per timepoint were six, four, four and four respectively. The viability measured on these timepoints does not capture any cells that have been dead before and have since been removed in the process. To quantify changes in cell number that may have influenced the live/dead staining results, we have evaluated cell density (number of total cells per mm^2^ of measured tissue) on day 0 and 28 (Appendix).

### 2.3 Histology

Cultures on days 0, seven, and 28 included four donors, day three or four included five donors. Samples were fixed in formalin and paraffin embedded. Samples were stained with Weigert's hematoxylin (ClinTech, Surrey, UK) followed by 0.4% Fast Green (Merck, Rahway, NJ, USA) and 0.125% Safranin-O (Merck, Rahway, NJ, USA).

Staining for type II collagen was performed. Endogenous peroxidase blocking with 3% H_2_0_2_ solution and 5% PBS/BSA was followed by antigen retrieval with pronase 1 mg/mL, (Sigma-Aldrich, St. Louis, MO, USA) and hyaluronidase 10 mg/mL (Sigma-Aldrich, St. Louis, MO, USA) at 37 °C, for 30 min each. Sections were incubated with mouse monoclonal antibody for type II collagen (DSHBII-II6B3), followed by incubation with a goat anti-mouse secondary antibody (Dako, Carpinteria, CA, USA) and staining with DAB solution. Sections were counterstained with Mayer's hematoxylin. An osteochondral allograft biopsy was used for a positive and negative control. The negative control was an isotype-matched control antibody (monoclonal mouse or rabbit IgG1, Dako (Carpinteria, CA, USA)). For type I and VI collagen, the same steps were followed, and sections were incubated with for type I collagen: rabbit monoclonal antibody (Abcam, Cambridge, UK) and secondary antibody (Brightvision, Immunologic, Duiven, the Netherlands) and for type VI collagen: mouse monoclonal antibody (DSHB 5C6 (Iowa City, IA, USA)) and goat anti-mouse secondary antibody (Dako, Carpinteria, CA, USA) and stained with DAB solution. A osteochondral biopsy was used for positive and negative control for type I collagen.

### 2.4 Proliferation

To assess cell proliferation, samples were cultured with 5-ethynyl-2′-deoxyuridine (EdU, Invitrogen, Carlsbad, CA, USA). EdU is a synthetic analog of thymidine which incorporates into newly synthesized DNA. EdU is added throughout the culture period of 28 days and cells that proliferate will incorporate the EdU into their DNA. After 28 days, sections were stained with Alexa-Fluor 555 and Hoechst 33342 using the Click-It EdU Imaging Kit (Invitrogen, Carlsbad, CA, USA) to visualize the proliferated cells. Proliferation was defined as percentage from total cells. Images were captured by Zeiss Zen Microscopy Software (RRID: SCR_013672) and analyzed using ImageJ software [20].

### 2.5 *Gelatinase activity* analysis

Cell-culture supernatant was collected throughout the culture period. Collected culture supernatant was loaded onto a 10% gelatin Zymogram gel and separated by SDS-PAGE. Collected supernatant was combined with 2x Tris-glycine SDS sample buffer. Supernatant volume was equalized for 40 μg protein. After electrophoresis, the Novex Zymogram renaturing buffer (Invitrogen, Carlsbad, CA, USA) was used at ambient temperature, followed by development in developing buffer, both for 30 min with gentle agitation. Lastly, the gel was incubated overnight at 37 °C in fresh developing buffer. Colloidal blue staining (Invitrogen, Carlsbad, CA, USA) was used to visualize the gelatinolytic activity. Band intensities correspond to 62 kDa for active MMP-2, 72 kDa for proMMP-2, 82 kDa for active MMP-9 and 92 kDa for proMMP-9.

### 2.6 Sulphated glycosaminoglycans (sGAG) analysis

sGAG release of four donors with multiple replicates was quantified using dimethylmethylene blue assay (Sigma-Aldrich, St. Louis, MO, USA) on cell-culture supernatants combined over 28 days of culture. The absorbances at 525 and 595 nm were subtracted, samples were compared to a standard curve of chondroitin Sulfate C (C4384 Sigma-Aldrich, St. Louis, MO, USA). sGAGs release could indicate loss of pre-existing sGAGs in the matrix or novel sGAG production.

### 2.7 Statistical analysis

Statistical analyses were performed in GraphPad Prism version 10.4.1 for Mac OS X (GraphPad Software, Boston, MA, USA, www.graphpad.com) and IBM SPSS Statistics version 29.0.2.0 (20) for Mac OS X (BM Corp., Armonk, N.Y., USA). Cell viability per group and over time, cell proliferation and sGAG concentration data were presented as estimated marginal means (EMM) and 95%-confidence interval (95%-CI). Statistical differences were evaluated using a generalized estimation equation. Standard deviation corresponding to EMM were displayed in the figures. Statistical differences were indicated with symbols in the figures and the figure legends. An overview of corresponding p-values and 95%-CIs for the cell viability, cell proliferation and sGAG concentration are shown in the Appendix Tables 1–2. Analysis was performed with an identity link and normal distribution, accounting for repeated measurements within donors using standard errors. Pairwise comparisons between all groups were conducted using EMM derived from the generalized estimation equation model. Bonferroni correction was applied to control for multiple testing across ten pairwise comparisons for cell viability and cell proliferation (between groups) and sGAG concentration, with statistical significance defined as p < 0.005 for comparisons between groups and p < 0.0025 for comparisons of cell viability over time to account for 20 pairwise comparisons.

B. Clinically used cartilage

### 2.8 Tissue collection

To evaluate cartilage used in clinical practice for autologous minced cartilage implantation, cartilage was collected from debrided cartilage defects treated with ACI and at loose body removal (both are considered medical waste or redundant material). This reflects the cartilage used for the clinical minced cartilage procedure (n = 12, male and female, aged 17–40). The tissue collection was performed according to the guideline “Human Tissue and Medical Research: Code of Conduct for responsible use” of the Dutch Federation of Medical Research Societies [21,22]. Wet weight was measured in mg after collection from the OR. Dry weight was measured in mg after lyophilizing the samples for 48 h. Water and dry weight ratios were calculated in percentages of wet weight. Collected data is shown per graph and each bar represents one patient.

### 2.9 sGAG and DNA content

Samples were digested overnight at 60 °C in a papain buffer (7.75 units/mL papain (Sigma-Aldrich), 0.2 M Na_2_H_2_PO_4_, 0.01 M EDTA_2_H_2_O, and 1.57 mg/mL cysteine HCl). The PicoGreen dsDNA quantification assay (ThermoFisher, Waltham, MA, USA) was used according to the manufacturer's instructions to determine DNA content of the cartilage samples. The excitation was set at 485 nm and the emission at 520 nm, results were compared to a standard curve of λDNA. A dimethylmethylene blue assay was used to quantify sGAGs as described above.

## 3 Results

A. Healthy cartilage samples

### 3.1 Viability of minced cartilage and isolated cells over time

On day 0, the mean viability was 56.1% (44.8–67.3) for chondral allograft biopsy, 61.1% (52.1–70) for manually minced and 50.1% (39.3–60.9) for arthroscopically minced cartilage. The mean viability for chondrons and chondrocytes on day 0 was 75.6% (63.9–87.3) and 76.6% (62.4–90.9) resp. On day 28, the chondral allograft biopsy had a mean viability of 72.4% (60.1–84.7), 79.8% (68.9–90.8) for manually minced cartilage, 56.6% (42.2–71.0) for arthroscopically minced cartilage, 93.7% (92.3–95.1) for chondrons and 94.0% (92.8–95.1) for chondrocytes. Of the donors tested on day 28, the chondral allograft biopsy on day 0 showed a mean viability of 68.7% (64.6–79.6). There were no statistical differences over time for all groups. (Fig. 1a). Representative images of the live/dead staining can be seen in Fig. 1b. Mean cell density decreased from day 0 to 28 for arthroscopically minced (700 → 164), chondrons (797 → 560) and chondrocytes (821 → 670) (Appendix).

**Fig. 1:**
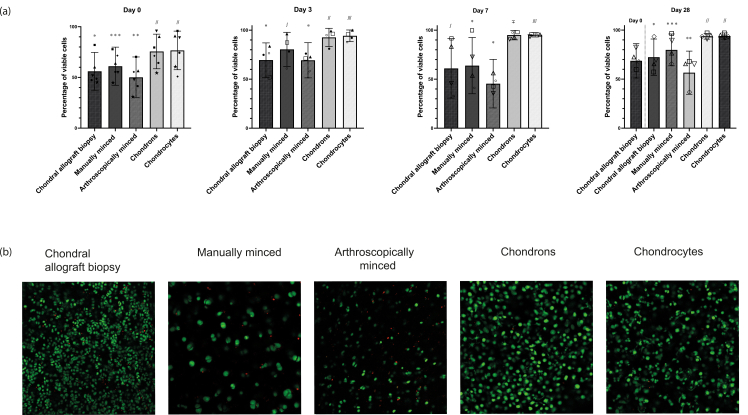
**Viability of minced cartilage and isolated cells.** 1a. Viability of minced cartilage and isolated chondrons and chondrocytes on day 0, three, seven, and 28. A biopsy from a chondral allograft, minced cartilage minced manually or arthroscopically and isolated chondrons and chondrocytes were cultured in fibrin gel and viability was determined at day 0, three, seven, and 28. Each point represents a donor with a minimum of 3 replicates per donor. Each donor is represented by a different symbol. Standard deviations are shown. A day 0 analysis is included for the donors included on day 28. A GEE analysis with a Bonferroni post-hoc correction for multiple testing between all groups was performed. All statistical significance for comparisons between groups including specific p-values and 95%-CI can be found in the appendix. Statistical differences are shown and marked as following: ∗ Significantly different from chondrons and chondrocytes; ∗∗ Significantly different from manually minced, chondrons and chondrocytes; ∗∗∗Significantly different from arthroscopically minced; ʃʃ Significantly different from chondral allograft biopsy and arthroscopically minced; ± Significantly different from chondrocytes; ʃʃʃ Significantly different from chondral allograft biopsy, arthroscopically and manually minced; ∓ Significantly different from arthroscopically and manually minced; 1b. All pictures are taken at 20x magnification. Images show live cells (green) and dead cells (red) for the five different groups on day 28. Images were combined in a Z-stack.

### 3.2 Histology

Safranin-O staining and type II collagen staining were positive in the chondral allograft biopsy, manually, and arthroscopically minced cartilage at day 0. Chondrons and chondrocytes demonstrated no GAG presence nor type II collagen presence at day 0. Type VI collagen was positive in all groups but the chondrons and chondrocytes at day 0.

**Fig. 2:**
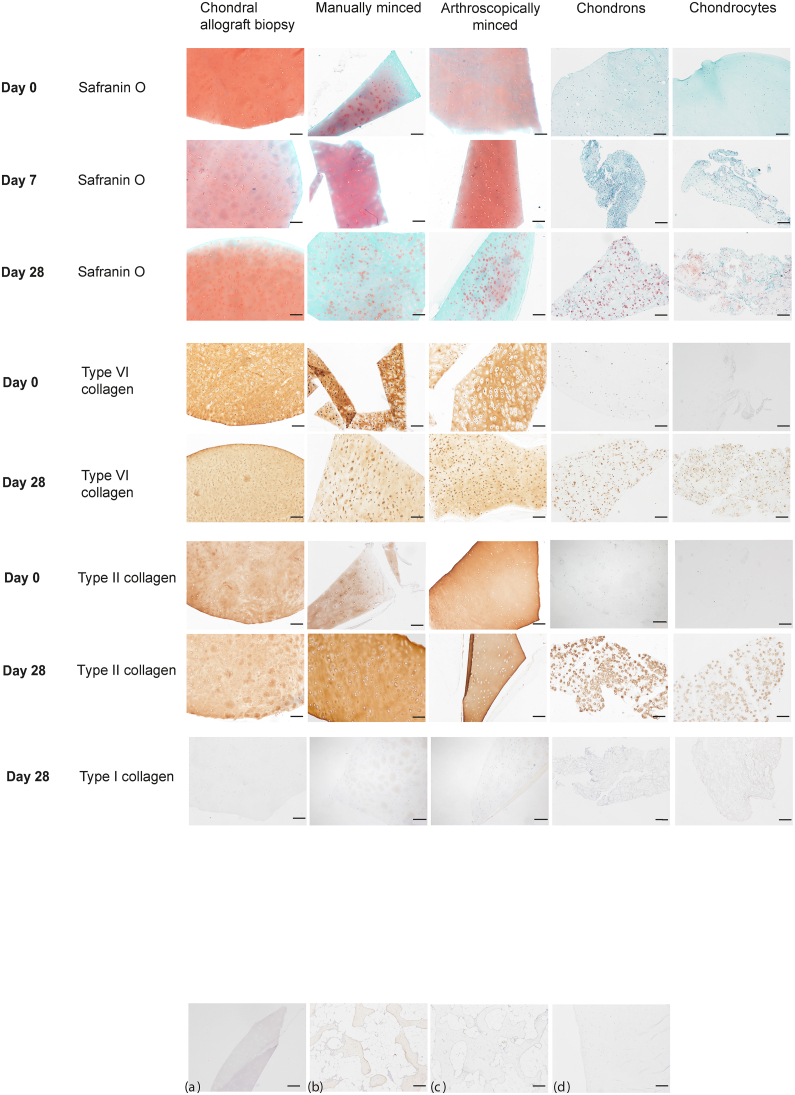
**Safranin-O/Fast Green staining, combined type II collagen and type I collagen staining and type VI collagen staining for different groups on day 0, seven and 28.** A biopsy from a chondral allograft, minced cartilage minced manually or arthroscopically and isolated chondrons and chondrocytes were cultured in fibrin gel and stained for GAGs with Safranin-O/Fast Green on day 0, seven, and 28. These groups were also stained with type II and VI collagen on day 0 and 28 and for type I collagen on day 28. All pictures with 200μ bar scale. Staining for Safranin-O/Fast Green, type I, II, and VI collagen (brown) are shown. (a) Image showing an osteochondral biopsy as negative control for type VI collagen. (b) Image showing an osteochondral biopsy as positive control for type I collagen (c) Image showing an osteochondral biopsy as negative control for type I (d) Image showing an osteochondral biopsy as a negative control for type II collagen. DAB: 3,3′-diaminobenzidine staining (brown).

On day 28, there were newly deposited GAGs, type II and VI collagen in the chondrons and chondrocytes. Manually and arthroscopically minced cartilage showed a decrease in Safranin-O from day 0 to day 28 while the chondral allograft biopsy demonstrated less Safranin-O depletion. Type VI collagen increased in the chondrocytes from day 0 to day 28. Type II and VI collagen staining intensity remained similar in the chondral allograft biopsy, manually, and arthroscopically minced cartilage between day 0 and day 28. Type I collagen was minimal in all groups at day 28 (Fig. 2).

### 3.3 Cell proliferation over time

**Fig. 3:**
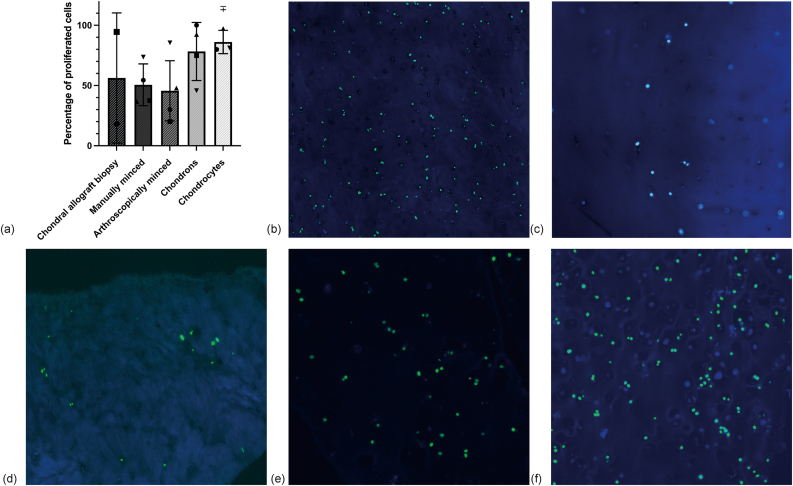
**Proliferation of minced cartilage and isolated cells on day 28.** A biopsy from a chondral allograft, minced cartilage minced manually or arthroscopically and isolated chondrons and chondrocytes were cultured in fibrin gel and proliferation was determined on day 28. (a) Graph showing the percentage of proliferated cells in minced cartilage and isolated cells on day 28. Each point represents a donor with a minimum of one replicate per donor. Each donor is represented by a different symbol. Standard deviations are shown. A GEE analysis with a Bonferroni post-hoc correction for multiple testing between all groups was performed. All statistical significance for comparisons between groups including specific p-values and 95%-CI can be found in the appendix. Statistical differences are shown and marked as following: The chondrocytes group was statistically significantly higher than the arthroscopically and manually minced group. Picture b through f shows proliferated cells (green) and all cells (blue). (b) Chondral allograft biopsy; (c) Manually minced cartilage; (d) Arthroscopically minced cartilage; (e) Chondrons; (f) Chondrocytes. Isolated cell groups show a homogenous distribution of proliferated cells. Minced cartilage groups show less proliferation on the edges in comparison to the chondral allograft biopsy. All pictures taken at 20x magnification.

The mean proliferation was 86.1% (77.2–95.1) in chondrocytes, 78.2% (57.8–98.7) in chondrons, 50.6% (35.8–65.3) for manually minced, 45.6% (26.3–64.9) for arthroscopically minced and 56.3% (3.3–109.2) for the chondral allograft group (Fig. 3a). The chondral allograft biopsy showed evenly distributed proliferated cells throughout the imaged biopsy (Fig. 3b), as did the cells in the chondrons and chondrocytes (Fig. 3e and f). Manually and arthroscopically minced cartilage mostly showed proliferation away from the edge of the minced tissue (Fig. 3c and d).

### 3.4 sGAG concentration in cell-culture supernatant and MMP-2 and MMP-9 zymography

**Fig. 4:**
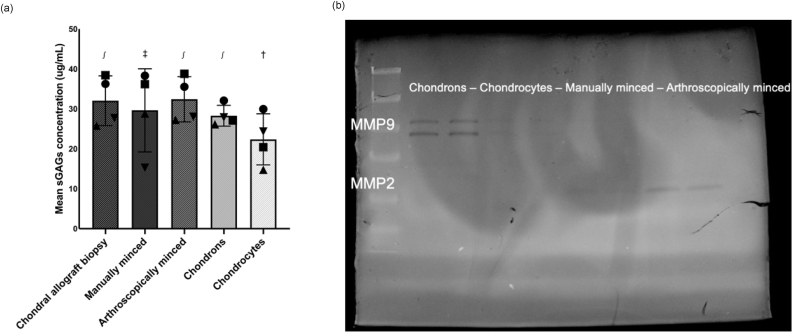
**Mean sGAG concentration in cell-culture supernatant and MMP-2 and MMP-9 zymography.** 4a. Mean sGAG concentration in cell-culture supernatant in different groups over 28 days. A biopsy from a chondral allograft, minced cartilage minced manually or arthroscopically and isolated chondrons and chondrocytes were cultured in fibrin gel and cell-culture supernatant was collected over 28 days and analyzed for sGAG concentration. Each bar represents the mean sGAG concentration in cell-culture supernatant in μg/mL with each point representing a donor with a minimum of 4 replicates. Each donor is represented by a different symbol. Standard deviation is shown. A GEE analysis with a Bonferroni post-hoc correction for multiple testing between all groups was performed. All statistical significance for comparisons between groups including specific p-values and 95%-CI can be found in the appendix. Statistical differences are shown and marked as following; †Statistically differs from chondral allograft biopsy, arthroscopically minced and chondrons; ‡ No significant differences; ʃ Statistically differs from chondrocytes. sGAG: sulphated glycosaminoglycans. 4b. Zymography of gelatinases in collected medium of different groups over 28 days. Cell-culture supernatant from cultures containing chondrons, chondrocytes, and the arthroscopically and manually minced cartilage was analyzed for matrix metalloproteinase 2 and 9. The first lane of the figure contains a ladder corresponding with different amounts of kDa.

The mean sGAG concentration (μg/mL) in the cell culture supernatant was 22.4 (16.9–27.8) for chondrocytes, 28.3 (26.1–30.5) for chondrons, 32.4 (27.6–37.2) for arthroscopically minced, 29.7 (20.8–38.5) for manually minced and 32.1 (26.8–37.4) for the chondral allograft biopsy (Fig. 4a).

**Fig. 5:**
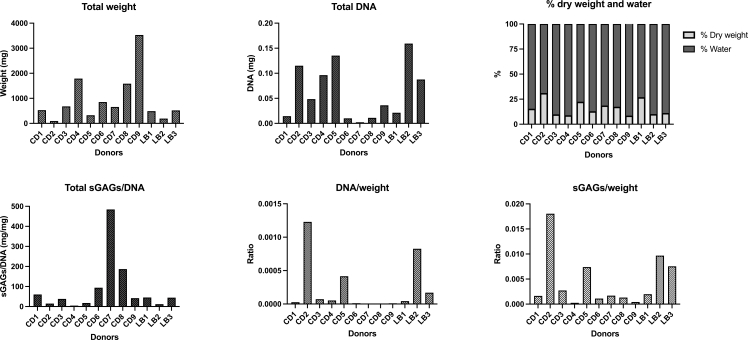
**Donor variability for total weight, total DNA, % dry weight and water, total sGAGs/DNA and DNA/weight.** Cartilage derived from two types of donors: cartilage defect debridement tissue from ACI; and cartilage from loose body removals was analyzed for weight, DNA and sGAGs on day 0. CD: cartilage defect; LB: loose body.

MMP-2 and MMP-9 activity was analyzed by gelatin zymography. A prominent band at 92 kDa and 82 kDa was observed in the chondrons, indicating proMMP-9 and active MMP-9. Lighter bands of pro-MMP-9 and active MMP-9 were present in the chondrocytes. A band was observed at 62 kDa, indicating active MMP-2, in the arthroscopically minced group. A lighter band of that size was present in the manually minced group. (Fig. 4b).

B. Analysis of clinically used cartilage

### 3.5 Donor variability of minced cartilage

Cartilage samples derived from debrided defects and loose bodies from clinical practice showed a high variability (more than 10-fold differences) in percentage of dry and wet weight, total DNA, DNA, sGAGs per weight, and total sGAGs per DNA (Fig. 5).

## 4 Discussion

This study compared isolated cell groups to different types of minced cartilage and a control tissue harvested from healthy donors in terms of viability, ECM deposition, and proliferation. It was demonstrated that the viability of isolated chondrons and chondrocytes were significantly higher than the cells in the minced cartilage groups over time. Furthermore, the isolated chondrons and chondrocytes deposited ECM over time while manually and arthroscopically minced cartilage groups demonstrated proteoglycan depletion.

In general, biopsies from osteochondral allografts were previously reported to demonstrate a viability between 2.2% and 90.9 % [23]. Similarly to literature, the donors used in our study show a high variability in viability. We included different donors for different timepoints. Of each donor, a control biopsy at day 0 was added to account for baseline differences in each donor. We did not find any differences in viability over time. Proliferation or complete disintegration of dead cells in which they no longer are capable of holding red dye, could be confounders. Although proliferation could be a confounder, based on cell density (Appendix-1A) and EdU proliferation (Fig. 3), it seemed to similarly affect the groups compared, therefore the confounding effect is thought to be small. Isolated cells had higher viability than manually minced cartilage after 28 days in culture. Arthroscopic mincing further decreased viability as demonstrated in our study and supported by prior findings using porcine [13], bovine [16], and tissue derived from fresh osteochondral allografts [12] when compared to manual mincing or controls. A significant decrease in viability of the device minced cartilage at day 0 which recovered after seven days was reported by Levinson et al. [15] while Evuarherhe et al. show the viability of the shaver minced group to increase from 0 to 42 days [12]. Although no reports on significance are made by the latter [12,15]. Contrastingly, our data did not demonstrate any significant differences from day 0 to 28 days. These differences may be attributed to the addition of fetal bovine serum to culture media which has a known positive impact on cell proliferation [24] and might have a positive effect on cell survival [25], although others show no effect [26]. The inclusion and comparison to isolated cells is a unique aspect of our study and highlights the importance in the clinical context as cell viability and cell dosage are crucial in the quality control of cell therapy and might have implications for the clinical outcomes of minced cartilage.

Moreover, GAG production and ECM deposition are essential for hyaline cartilage formation and necessary for the repair of cartilage defects. Our findings showed both chondrons and chondrocytes deposit sGAGs and produce type II collagen from day 0 to 28, the chondral allograft biopsy group remained stable while both minced cartilage groups showed proteoglycan loss over time on histology. Our findings are supported by significantly increased sGAGs in cell-culture supernatant of the arthroscopically minced cartilage group compared to chondrocytes. The allograft biopsy also shows significantly increased sGAGs in the cell culture supernatant compared to chondrocytes, however, it is important to highlight that the chondral allograft biopsies are larger and data are not normalised to weight. Similarly, another study showed minced cartilage derived from osteochondral allografts minced with an arthroscopic shaver to have less Safranin-O staining after 42 days of culture compared to the control explant and there appeared to be loss of proteoglycans in the minced group from day 0 to 42 [12]. Importantly, while there is loss of ECM components in minced cartilage, there is still ECM present. The functionality of this ECM is unclear, as it is unaligned and collagen turnover and remodelling is minimal in adult tissues [27].

While previous studies have reported positive Safranin-O staining and chondrocyte migration from osteoarthritic cartilage fragments after culture in growth factor-enriched media [11], our findings demonstrated a progressive loss of proteoglycans over time in minced cartilage groups, mostly pronounced between day seven and 28. Similar to our results, limited Safranin-O staining and positive type II collagen staining was shown in minced osteoarthritic cartilage after 28 days by Levinson et al. [15] Additionally comparable to our findings, positive GAGs in both groups at 1 week were found by Ossendorff et al. [17] In contrast to our results, no production of GAGs in isolated chondrocytes compared to minced cartilage derived from osteoarthritic tissue was reported by Tsuyuguchi et al. [18] The lower GAG production could be attributed to the addition of serum to the culture media instead of usual chondropermissive or chondrogenic media that are commonly serum free [28]. Our findings suggest a potential link between higher cell viability and GAG deposition, similar to positive correlations between cell viability and gross, histological and GAG content assessments as seen in osteochondral allograft transplantation [29], which may have implications for optimizing minced cartilage repair strategies.

Furthermore, a more intense MMP-9 activity was found in chondrons and chondrocytes, compared to minced cartilage. MMP-2 was highest in the arthroscopically and manually minced groups. Interestingly, our current study found a higher MMP-9 in chondrons compared to chondrocytes, in contrast to a prior study that showed higher MMP-9 in chondrocytes. They suggested this could be the result of altered cell-ECM interaction caused by the removal of the native pericellular matrix [30,31]. While prior research shows MMP-9 to have a role in cartilage degradation [32], MMP-9 was also suggested to have a positive role in chondrogenesis and ECM production by Kondo et al. [33] This is in line with our current study that shows ECM formation in chondrocytes over 28 days. Prior research has shown that MMP-2 is constitutively expressed in adult cartilage and has been shown to be upregulated in arthritic cartilage [32]. The increased MMP-2 in arthroscopically minced cartilage correlates with proteoglycan loss between day 0 and 28, potentially indicating tissue proteoglycan depletion.

Lastly, minced cartilage used in clinical practice is often harvested from debrided defects or loose bodies. Existing clinical evidence is limited to two RCTs (including 29 [10] and 88 patients [9]) and retrospective studies with variable outcomes and low patient numbers (≤30 patients per comparative group), all in the absence of quality control [[34], [35], [36]]. We found a high variability between clinical samples with a more than 10-fold differences. Prior research has shown differences in sGAGs, DNA, and weight between cartilage derived from different areas in the knee [14] as well as differences in proteoglycan content between healthy and osteoarthritic cartilage [37]. The high variability could have implications for clinical practice, as baseline tissue differences combined with differences resulting from different mincing techniques could impact outcomes, and therefore these data should be captured in clinical applications.

### 4.1 Limitations

First, this study was performed *in* vitro using cartilage harvested from healthy human osteochondral allografts, which were stored up to a maximum of 21 days before the start of this study. We demonstrated that quality of the tissue declines over time, although this is mainly true for the minced cartilage groups and did not similarly affect controls between day 0 and day 28. Storage time varied between 9 and 21 days and the effect on the loss of proteoglycans in the 28 days following is uncertain. However, all groups were derived from the same osteochondral allografts and are thus affected to the same extent. In addition, the age range of the allograft tissue donors was 13–32 years which only partially represents the patient population that suffers from cartilage defects, as the age range of the clinical tissue was 17–40 years. Furthermore, we did not apply any mechanical loading which could have had a positive effect on ECM. Another limitation of this study is that the live/dead assay showcases membrane integrity at a specific moment. This could potentially overestimate viability if dead cells have not yet suffered significant membrane degradation or underestimate viability if live cells have localized permeability defects, both of which are an inherent limitation of live/dead staining [38]. However, it was previously demonstrated that permeable cells do not stain positive (live) for Calcein AM [39] and that progressive increase of plasma membrane permeability promotes intracellular esterase activity live with Calcein AM [40]. Additionally, sGAG concentration was measured in the cell-culture supernatant which on its own cannot distinguish between new sGAG formation and release of pre-existing sGAGs from the matrix. Therefore, we followed GAG content over time using histology. We have not corrected the sGAGs release for weight or volume of the constructs, which could result in an underrepresentation of sGAG release in the isolated cell gels that are smaller. We did not perform quantitative analysis of the sGAG content in the samples as new production would be overshadowed by already present sGAGs. Novel labelling methods can differentiate between pre-existing sGAGs, and novel matrix produced and could be added in further research [[41], [42], [43]].

## 5 Conclusion

This study shows manually and arthroscopically minced cartilage have lower viability as compared to isolated cells, that have consistently high viability. Furthermore, minced cartilage has full ECM at the start of cultures that is subsequently partially depleted over 4 weeks, whereas isolated cells are completely depleted of ECM at the start of cultures and deposit proteoglycans and type II collagen over time. Cartilage tissue typically used for minced cartilage varies more than 10-fold in cell density and proteoglycan content. As minced cartilage is currently not quality controlled, the consequences of these variations on clinical treatment outcomes are unknown [9,10,[34], [35], [36]].Our findings, together with the scarcity of clinical trials and absence of quality control, call for caution in the implementation of minced cartilage as standard of care. There is a responsibility for the scientific and clinical field to systematically and thoroughly evaluate the effect of tissue and cell quality on clinical outcomes.

## Author contributions

DS was involved in data acquisition, statistical and histological analysis, data presentation and interpretation, writing of the original draft. CVN was involved in the conception and design of the study, supervision, review and editing of the manuscript. KLL was involved in data acquisition and review and editing of the manuscript. LAV was involved in the conception and design of the study, supervision, review and editing of the manuscript. AJK was involved in data acquisition by providing clinical samples for the study to be possible and was involved in review and editing of the manuscript. RJHC was involved the conception and design of the study, in supervision and review and editing of the manuscript. DBFS was involved in data acquisition by providing clinical samples for the study to be possible and was involved in the conception and design of the study, supervision, review and editing of the manuscript. JVK was involved in the conception and design of the study, conduction of experiments and data acquisition, supervision, review and editing of the manuscript. All authors read and approved the final manuscript.

## Statements

This research was performed at the Mayo Clinic (Rochester, Minnesota, United States of America) and the University Medical Center Utrecht (Utrecht, the Netherlands).

## Declaration of funding

We would like to acknowledge the Quattrone, Sachdev and the Hofvijverkring funds for supporting our department and laboratory. The Hofvijverkring is a non-profit aiming to promote research in Utrecht by supporting researchers through education grants and travel funds and has supported our research through travel funds. The Quattrone and Sachdev funds supported our research through education and travel support as well as laboratory support. All final decision regarding this study remained independent of the funders.

## Conflict of interest statement

- DS declares no potential conflicts of interest.
- CVN declares no potential conflicts of interest.
- KLL declares no potential conflicts of interest.
- LAV reports the following disclosures:
  • LAV is employed by and has stock options in Xintela AB
  • Board of Directors or committee member: International Cartilage Regeneration & Joint Preservation Society.
  • Editorial or governing board: Journal of Cartilage & Joint Preservation
- AJK reports potential conflicts of interest:
  • American Journal of Sports Medicine: Editorial or governing board
  • Arthrex, Inc: IP royalties; Paid consultant
  • Arthroscopy Association of North America: Board or committee member
  • International Cartilage Repair Society: Board or committee member
  • Springer: Editorial or governing board
- RJHC declares no potential conflicts of interest.
- DBFS reports the following disclosures:
  • MTF: Clinical advisory board member
  • JRF: Research support
  • Arthrex: Research support
  • ReLive Biotechnology: Consultant
  • Phoenix Kinetix: Consultant
  • Medacta: Consultant
  • Smith & Nephew: Consultant
- JVK declares no potential conflicts of interest.

## Figures and Tables

**Fig. 1:**
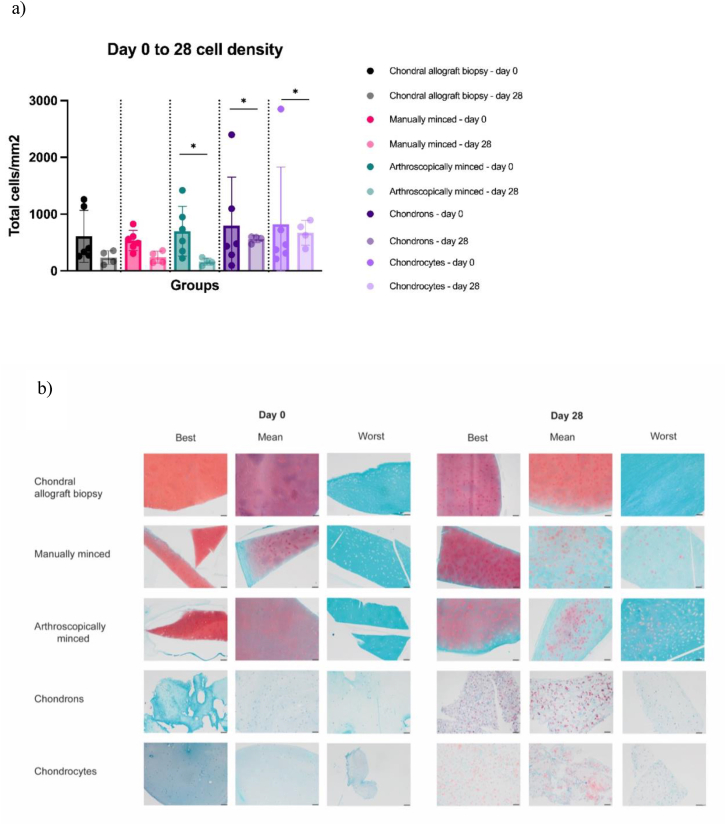
**A) Cell density on day 0 and day 28 for different groups.** A biopsy from a chondral allograft, minced cartilage minced manually or arthroscopically and isolated chondrons and chondrocytes were cultured in fibrin gel and imaged for live/dead analyses. The total number of cells present have been calculated as well as the area of the tissue in which the cells were present to calculate cell density (total cells/mm^2^). There is a significant decrease in cell density over time in the arthroscopically minced, chondrons and chondrocytes group. Each bar represents the mean cell density with each point depicting a donor with a minimum of 4 replicates. **B) Best, mean and worst overview of Safranin-O/Fast green staining on day 0 and day 28 for different groups.** A biopsy from a chondral allograft, minced cartilage minced manually or arthroscopically and isolated chondrons and chondrocytes were cultured in fibrin gel and stained for GAGs with Safranin-O/Fast Green on day 0 and 28. All pictures with 100μ bar scale. On both day 0 as day 28 GAG content varies between and among the groups depicted as best (most amount of GAG content) to worst (least amount of GAG content). The chondrons and chondrocytes show no GAG content on day 0 and show clear GAG deposition on day 28, depicted as best (most amount of GAG deposition) to worst (least amount of GAG deposition).

**Table 1:** Pairwise comparisons of estimated marginal means including significance and 95% confidence interval, subanalyses for cell viability, cell proliferation and sGAG concentration per different timepoint are shown separately below.

Pairwise comparisons of estimated marginal means between different groups with significance and 95% confidence interval for cell viability on day 0.				
Group	Compared with group	Significance	95% Wald Confidence Interval for Difference	
			Lower	Upper
**Chondrons**	Chondrocytes	0.848	−11.6986	9.6168
**Arthroscopically minced cartilage**	Chondrocytes	**<0.001**∗	−39.1148	−13.9597
	Chondrons	**<0.001**∗	−36.1588	−14.8338
**Manually minced cartilage**	Chondrocytes	0.034	−29.9614	−1.1954
	Chondrons	0.016	−26.3118	−2.7632
	Arthroscopically minced cartilage	**<0.001**∗	7.7865	14.1312
**Chondral allograft biopsy**	Chondrocytes	**<0.001**∗	−30.8412	−10.3265
	Chondrons	**0.002**∗	−31.6873	−7.3985
	Arthroscopically minced cartilage	0.028	0.6359	11.2710
	Manually minced cartilage	0.156	−11.9175	1.9066
*Pairwise comparisons of estimated marginal means between different groups with significance and 95% confidence interval for****cell viability on day 3.***				

**Chondrons**	Chondrocytes	0.593	−7.7524	4.4310
**Arthroscopically minced cartilage**	Chondrocytes	**<0.001**∗	−30.5776	−19.4047
	Chondrons	**<0.001**∗	−34.9327	−11.7282
**Manually minced cartilage**	Chondrocytes	**0.002**∗	−22.7271	−4.9587
	Chondrons	0.057	−24.7334	0.3689
	Arthroscopically minced cartilage	0.012	2.4899	19.8065
**Chondral allograft biopsy**	Chondrocytes	**<0.001**∗	−38.4555	−11.1480
	Chondrons	**0.003**∗	−38.5590	−7.7232
	Arthroscopically minced cartilage	0.980	−14.9288	15.3075
	Manually minced cartilage	0.286	−31.0904	9.1728
*Pairwise comparisons of estimated marginal means between different groups with significance and 95% confidence interval for****cell viability on day 7.***				

**Chondrons**	Chondrocytes	0.727	−2.3018	1.6066
**Arthroscopically minced cartilage**	Chondrocytes	**<0.001**∗	−56.9926	−42.7798
	Chondrons	**<0.001**∗	−57.4192	−41.6579
**Manually minced cartilage**	Chondrocytes	**0.001**∗	−50.8665	−12.1173
	Chondrons	**0.002**∗	−50.5210	−11.7676
	Arthroscopically minced cartilage	0.139	−5.9590	42.7475
**Chondral allograft biopsy**	Chondrocytes	**0.004**∗	−57.9820	−10.7362
	Chondrons	0.006	−58.4375	−9.5854
	Arthroscopically minced cartilage	0.165	−6.3796	37.4338
	Manually minced cartilage	0.798	−24.8230	19.0887
*Pairwise comparisons of estimated marginal means between different groups with significance and 95% confidence interval for****cell viability on day 28***.				

**Chondrons**	Chondrocytes	0.253	−0.6485	0.1704
**Arthroscopically minced cartilage**	Chondrocytes	**<0.001**∗	−51.4573	−23.3111
	Chondrons	**<0.001**∗	−51.4285	−22.8618
**Manually minced cartilage**	Chondrocytes	0.009	−24.7718	−3.4682
	Chondrons	0.013	−24.8194	−2.9425
	Arthroscopically minced cartilage	**<0.001**∗	16.0080	30.5204
**Chondral allograft biopsy**	Chondrocytes	**0.001**∗	−34.5713	−8.5609
	Chondrons	**0.001**∗	−34.2912	−8.3629
	Arthroscopically minced cartilage	0.240	−10.5531	42.1893
	Manually minced cartilage	0.509	−29.5709	14.6787
*Pairwise comparisons of estimated marginal means between different groups with significance and 95% confidence interval for****cell proliferation on day 28.***				

**Chondral allograft biopsy**	Manually minced cartilage	0.820	−43.2856	54.6499
	Arthroscopically minced cartilage	0.697	−43.0204	64.3321
	Chondrons	0.369	−69.9442	25.9533
	Chondrocytes	0.299	−86.3605	26.5741
**Manually minced cartilage**	Arthroscopically minced cartilage	0.430	−7.3907	17.3381
	Chondrons	0.091	−59.7500	4.3947
	Chondrocytes	**0.002**∗	−57.6233	−13.5274
**Arthroscopically minced cartilage**	Chondrons	0.083	−69.5339	4.2313
	Chondrocytes	**<.001**∗	−62.6165	−18.4816
**Chondrons**	Chondrocytes	0.428	−27.4288	11.6334

*Pairwise comparisons of estimated marginal means between different groups with significance and 95% confidence interval for****sGAG concentration over 28 days.***				

**Chondral allograft biopsy**	Manually minced cartilage	0.435	−3.6134	8.3964
	Arthroscopically minced cartilage	0.352	−1.1199	0.3990
	Chondrons	0.115	−0.9085	8.4186
	Chondrocytes	**<0.001**∗	4.2574	15.1128
**Manually minced cartilage**	Arthroscopically minced cartilage	0.368	−8.7459	3.2420
	Chondrons	0.745	−6.8453	9.5724
	Chondrocytes	0.138	−2.3394	16.9266
**Arthroscopically minced cartilage**	Chondrons	0.071	−0.3479	8.5788
	Chondrocytes	**<0.001**∗	4.3396	15.7515
**Chondrons**	Chondrocytes	**<0.001**∗	2.4392	9.4209

∗ Significance is set on <0.005 to adjust for multiple comparisons using Bonferroni. Duplicate comparisons are removed.

**Table 2:** Pairwise comparisons of estimated marginal means for different groups over time with significance and 95% confidence interval for cell viability.

Timepoint	Compared with timepoint	Significance	95% Wald Confidence interval for difference	
			Lower	Upper
**Chondral allograft biopsy**				
**Day 28**	0	.054	−0.3150	32.9994
**Manually minced cartilage**				
**Day 28**	0	.009	4.6231	32.9427
**Arthroscopically minced cartilage**				
**Day 28**	0	.481	−11.5433	24.4984
**Chondrons**				
**Day 28**	0	.003	6.3001	29.9525
**Chondrocytes**				
**Day 28**	0	.017	3.0396	31.6093

∗Significance is set on <0.0025 to adjust for multiple comparisons using Bonferroni.
